# Toward a Mechanistic Understanding of Poly- and Perfluoroalkylated Substances and Cancer

**DOI:** 10.3390/cancers14122919

**Published:** 2022-06-14

**Authors:** Raya I. Boyd, Saeed Ahmad, Ratnakar Singh, Zeeshan Fazal, Gail S. Prins, Zeynep Madak Erdogan, Joseph Irudayaraj, Michael J. Spinella

**Affiliations:** 1 Department of Comparative Biosciences, University of Illinois, Urbana-Champaign, Urbana, IL 61802, USA; rayaib2@illinois.edu (R.I.B.); rsingh02@illinois.edu (R.S.); fazal2@illinois.edu (Z.F.); 2 Department of Bioengineering, University of Illinois, Urbana-Champaign, Urbana, IL 61801, USA; saeeda2@illinois.edu (S.A.); jirudaya@illinois.edu (J.I.); 3 Departments of Urology, Pathology and Physiology, College of Medicine, Chicago Center for Health and Environment, University of Illinois Chicago, Chicago, IL 60612, USA; gprins@uic.edu; 4 Department of Food Science and Human Nutrition, Division of Nutritional Sciences, University of Illinois Urbana-Champaign, Urbana, IL 61801, USA; zmadake2@illinois.edu; 5 Institute of Genomic Biology, University of Illinois, Urbana-Champaign, Urbana, IL 61801, USA; 6 Beckman Institute of Technology, University of Illinois, Urbana-Champaign, Urbana, IL 61801, USA; 7 Cancer Center at Illinois, University of Illinois, Urbana-Champaign, Urbana, IL 61801, USA

**Keywords:** PFOA, PFOS, testicular cancer, prostate cancer, epigenetics, metabolomic

## Abstract

**Simple Summary:**

Poly- and perfluoroalkylated substances (PFAS) are industrial chemicals found in many household products that persist in the environment. While several excellent review articles exist on the potential harmful effects of PFAS, there are few focused on cancer. This concise and streamlined mini-review focuses on summarizing molecular mechanisms related to the potential cancer-promoting properties of PFAS. This review organizes and interprets the vast primary PFAS cancer biology literature and provides a coherent, unified, and digestible model of the molecular mechanisms that potentially explains PFAS cancer promotion.

**Abstract:**

Poly- and perfluoroalkylated substances (PFAS) are chemicals that persist and bioaccumulate in the environment and are found in nearly all human populations through several routes of exposure. Human occupational and community exposure to PFAS has been associated with several cancers, including cancers of the kidney, testis, prostate, and liver. While evidence suggests that PFAS are not directly mutagenic, many diverse mechanisms of carcinogenicity have been proposed. In this mini-review, we organize these mechanisms into three major proposed pathways of PFAS action—metabolism, endocrine disruption, and epigenetic perturbation—and discuss how these distinct but interdependent pathways may explain many of the proposed pro-carcinogenic effects of the PFAS class of environmental contaminants. Notably, each of the pathways is predicted to be highly sensitive to the dose and window of exposure which may, in part, explain the variable epidemiologic and experimental evidence linking PFAS and cancer. We highlight testicular and prostate cancer as models to validate this concept.

## 1. Introduction

**Figure 1:**
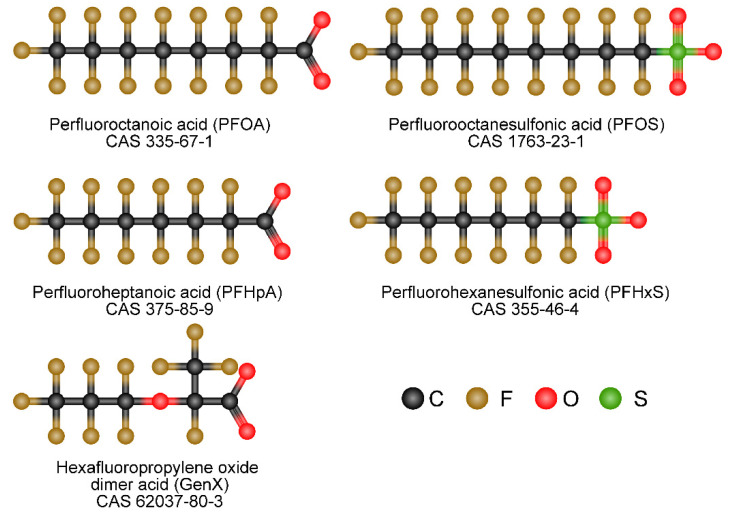
Chemical structures of Poly- and perfluoroalkylated substances. Structures are based on 2D structures from PubChem accessed 5 June 2022 and assembled in Chem-space.com accessed 5 June 2022.

Poly- and perfluoroalkylated substances (PFAS) are a class of chemicals used in many industrial and consumer products to resist heat, stains, water, and grease (Figure 1) [1]. Examples include Teflon, coatings on fast food wrappers and nonstick pans, floor polish, carpets, furniture fabrics, firefighting foams, clothing treatments, and many others [1,2]. The manufacture, application, and disposal of fluorochemicals, since the 1940s, have led to worldwide pollution of PFAS, which affects not only water sources, but also food production, soil, runoff, and groundwater sources [3,4]. Fire suppression activities are also a major contributor to PFAS contamination [5]. It has been estimated that 99% of Americans have PFAS chemicals in their bodies [6]. In addition, perfluorochemicals are resistant to biodegradation, resulting in long residence times in the environment and body, with a human serum half-life for some PFAS of up to 2–5 years [6,7]. Several communities near chemical plants that manufacture PFAS have documented serum levels over 50-fold higher than the general population due to contaminated drinking water [8,9,10]. Human epidemiological studies have found that exposure to two legacy PFAS, perfluorooctanoic acid (PFOA) and perfluorooctanesulfonic acid (PFOS), is associated with various negative health outcomes, including elevated cholesterol and liver enzyme levels, thyroid disorders, pregnancy-induced hypertension, preeclampsia, and cancer [11,12,13,14,15,16]. PFOA has been classified as a group 2B carcinogen, and the EPA has classified PFOA, PFOS, and the newer shorter-chain PFAS, hexafluoropropylene oxide GenX, as having carcinogenic potential [17,18,19].

Multiple epidemiologic studies have been supportive but not definitive in linking PFAS exposure to cancer, including cancers of the kidney, testis, prostate, liver, breast, pancreas, bladder, and non-Hodgkin’s lymphoma [20,21,22,23,24,25,26,27,28]. However, other studies have shown inconsistent or negative correlations [29,30,31]. This may be due to differences in study design, difficulties in modeling PFAS exposures, and differences in the dosages and windows of exposure to PFAS, which may be critical for a variety of cancers. A scoping review of 16 cohort studies, 10 case-control studies, 1 cross-sectional study, and 1 ecological study concluded that the cancer sites with the most compelling evidence for an association with PFAS exposure across studies were kidney and testicular cancers, followed by prostate cancer [14]. A separate meta-analysis, focused on kidney and testicular cancer, indicated a significant increase in cancer risk per 10 ng/mL increase in serum PFOA for kidney and testicular cancer, and that these associations were most likely causal [32]. In addition, rodent studies have shown that PFOA, PFOS, and GenX can increase the rate of Leydig cell adenoma, pancreatic acinar cell adenoma, hepatocellular adenoma and carcinoma, and thyroid adenoma, although the human relevance of these findings has been called into question [33,34,35,36]. The health concerns related to PFAS have attracted much attention from the public and the scientific community. Despite past efforts, the mechanisms of action of PFAS, especially in relation to cancer, are poorly understood. Here, we review and synthesize the major proposed cancer mechanisms related to PFAS exposure.

## 2. Potential Mechanisms of PFAS Carcinogenesis

Unlike known carcinogens such as benzo(a)pyrene and UV light that are genotoxic due to direct damage to DNA, there is little evidence that PFAS are direct mutagens or deregulators of DNA repair or genomic stability [37,38,39]. However, at high concentrations, PFAS have been demonstrated to damage DNA via reactive oxygen species generation [40,41]. It is unclear if this mechanism is relevant for typical levels of human PFAS exposure. In contrast, most of the evidence for PFAS-mediated effects has focused on epigenetics, transcription, cellular metabolism, and endocrine effects [11,12,37,42,43,44].

### 2.1. Metabolism

Metabolic plasticity is one of the hallmarks of cancer [45]. PFAS exposure causes numerous metabolic alterations, through both PPAR-dependent and -independent mechanisms in the liver and other tissues [11,42]. Structurally, PFAS resemble fatty acids (FAs) and there is evidence that PFAS can act as ligands for peroxisome proliferator-activated receptors (PPARs) [46,47]. PPARs are transcription factors with many biological effects beyond their canonical role in controlling lipid and glucose metabolism [48]. Hence, activation of PPARs is an attractive mechanism to explain many of the biological effects of PFAS. The activation of PPARα has been extensively studied as a mechanism of PFAS-mediated liver toxicities, including fibrosis, cirrhosis, steatosis, non-alcoholic fatty acid liver disease, and liver cancer [49,50,51,52]. Similarly, the PFAS activation of PPARs has also been proposed to mediate dyslipidemia (especially high cholesterol), insulin resistance, adipogenesis, and several cancers, including colon, breast, and prostate cancer [11,42,53,54,55,56,57]. Likely related again to a structural similarity with FAs, PFAS are known to accumulate in the liver and have been proposed as altering FA metabolism by binding to FA transporters and metabolic enzymes [11,42]. In contrast to PFAS activation of PPARs, there is less evidence for direct activation by PFAS of other metabolic and xenobiotic nuclear receptors that respond to FAs, including liver X (LXR), farnesoid X (FXR), constitutive androstane (CAR), and pregnane X (PXR). Since altered metabolism is a key feature of the cancer phenotype, the alteration of metabolic regulators such as PPARs offers an attractive mechanism for the proposed pro-carcinogenetic actions of PFAS [45]. Another mechanism related to FA mimicry is the proposed direct effect of PFAS on regulating cell membrane fluidity [58,59]. Published studies demonstrate a central role for PPARα signaling in PFOA/PFOS-induced liver and kidney carcinogenesis [21,60]. In addition, an important role for fatty acid metabolism has been proposed for other cancers including breast, prostate, and colon cancer [61,62,63].

PFOA has been proposed to increase the risk of metabolic syndrome in humans [57]. PFAS alter the hepatic metabolism, with alterations in amino acid biogenesis and the Krebs cycle [64]. In addition, the upregulation of enzymes involved in β-oxidation has been reported upon PFOS exposure [65]. PFOS also induced high peroxisome, endoplasmic reticulum, mitochondria, and membrane protein levels, and deregulated lipid and amino acid metabolism [66,67]. Prenatal exposure to PFAS can contribute to pediatric liver toxicity [68]. A study of 1105 mother-child pairs that assessed multiple PFAS in maternal blood found higher liver enzyme levels of alanine aminotransferase, aspartate aminotransferase and gamma-glutamyl transferase [68]. Furthermore, PFAS levels were associated with alterations in serum amino acid levels in children [69]. In a study of male Chinese subjects, six PFAS were associated with metabolic serum changes associated with oxidative stress [70]. Metabolic stress, as evidenced by metabolites of oxidative DNA damage and lipid peroxidation, has also been documented for both animal and cell line studies for a number of PFAS compounds [54,70]. An additional study of targeted metabolomics found perturbations in branched-chain and aromatic amino acid biosynthesis and glycerophospholipid metabolism and a link between PFAS and increased risk of non-alcoholic steatohepatitis in children [68]. Rodent experiments have shown that early and prenatal PFAS is associated with liver injury in offspring [71,72].

**Figure 2:**
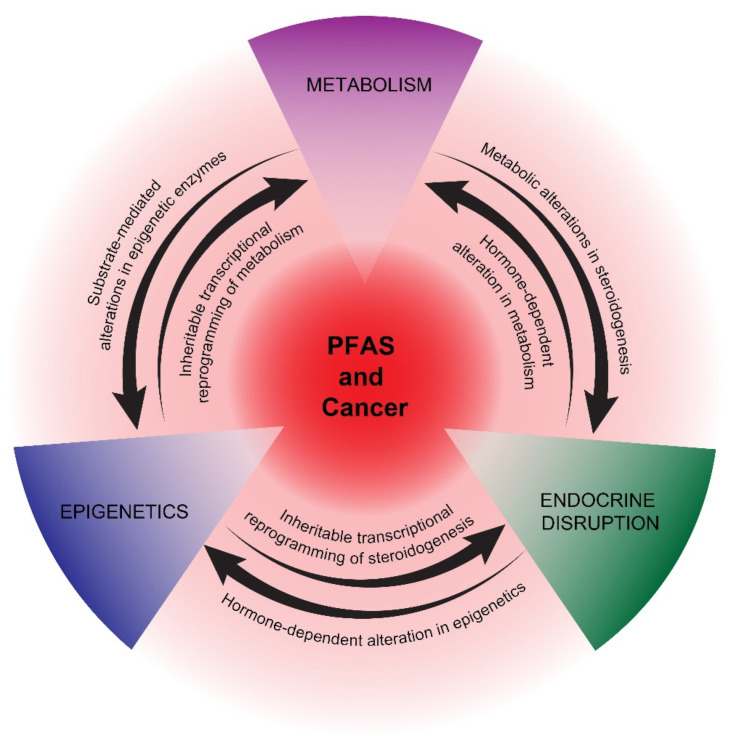
Proposed mechanisms of potential PFAS cancer promotion. PPAR-dependent and -independent reprogramming of metabolism, epigenetics, and endocrine disruption are represented as interconnecting, mutually reinforcing pathways of potential PFAS tumor promotion. The precise details of how PFAS influences these pathways are still uncertain, as is the impact of other proposed PFAS mechanisms, including immunosuppression and oxidative stress.

In summary, the activation of PPARs and associated metabolic perturbations, especially in the liver, is one of the most studied mechanisms of PFAS actions. The recent appreciation that many cancers are driven and sustained by metabolic reprogramming underscores the potential importance of this pathway in studying the proposed pro-carcinogenic effects of PFAS. How metabolic reprogramming at the hepatic and cancer cell/cancer progenitor cell level cross-talks with epigenetic and endocrine reprogramming is a key area of future research for understanding the potential carcinogenicity of PFAS (Figure 2).

### 2.2. Endocrine Disruption

PFAS cross the placenta and concentrate in breast milk; thus, exposure to the developing fetus and infant occurs [73,74]. PFAS are known to have endocrine-disrupting properties [75,76]. There are reports of adverse reproductive health and decreased fecundity linked to PFAS exposure [77,78]. Human semen quality has decreased over the last several decades. This time period coincides with the rise in production of endocrine-disrupting chemicals (EDCs), and PFAS have been associated with infertility in male mice and subfertility in female mice [79,80]. In several studies, estrogenic and anti-androgen activities were observed for a number of PFAS compounds [81,82,83,84]. There is evidence that PFAS exposure is associated with decreased testosterone and poor sperm quality and numbers in humans [78,85]. For example, in a Japanese study, in utero PFOA and PFOS exposure was associated with decreased testosterone in male neonates [86]. In addition to in human studies, in rodents, PFAS have been observed to alter testosterone and estrogen levels, and were associated with impaired spermatogenesis and steroidogenesis and reduced sperm quality [81,82,83,84], although some inconsistent findings also exist [87]. In female rodents, PFOA alters mammary development [88,89]. PFOA has been associated with changes in the uterus and the reproductive health of female mice [90].

Several cancers associated with PFAS are hormone-dependent, including prostate and breast cancer, or have an etiology closely associated with endocrine disruption, as in testicular cancer [22,23,24,25,26,27,28]. In addition, endometrial cancer has been associated with endocrine disruption [91]. There is evidence that PFAS can alter endocrine hormone levels, potentially leading to disrupted reproductive health, especially with neonatal or pubertal exposure [92,93,94]. A major proposed mechanism of EDCs, in general, is their binding to nuclear receptors [95]. While there is strong evidence supporting the direct activation of PPARs, there is less evidence that PFAS directly activate endocrine receptors, including estrogen (ER) and androgen receptors (AR). Hence, the mechanism of endocrine disruption mediated by PFAS remains unclear, suggesting that indirect mechanisms, including epigenetic and/or metabolic reprogramming, may play roles in disrupting the production and secretion of endocrine hormones during critical windows of exposure [44,96] (Figure 2). In turn, early-life exposure to EDCs has been associated with epigenetic reprogramming that manifests later in life [97].

### 2.3. Epigenetics

Despite the likelihood that non-mutagenic, epigenetic pathways play a major role in PFAS biological effects, studies have been sparse, and these have mainly focused on DNA methylation. PFAS have been shown to be associated with both hypomethylation and hypermethylation in genome-wide and gene-specific molecular epidemiology studies [98,99,100,101,102,103,104,105]. PFAS levels have also been linked to decreased and differential DNA methylation in infants [102,103,104]. For example, reduced insulin-like growth factor methylation in cord blood was observed with prenatal PFOA exposure [104]. Another study reported that PFAS exposure was associated with increased long interspersed nuclear element-1 methylation [99]. Associations between PFAS exposures and altered methylation, either genome-wide or at specific loci, have been described in limited in vitro and animal studies, including early life exposures in rodents [106,107,108,109,110,111,112,113,114,115,116]. One study revealed PFOA-mediated hypomethylation of the glutathione-S-transferase Pi gene in liver cells [108]. Significant alterations in DNA methylation have been reported in vitro in HepG2 cells and in vivo in mouse kidney and liver tissues [111,112,113]. Globally, DNA methylation is altered during PFOS-induced fat cell differentiation [109]. Additionally, recent studies have reported PFAS-mediated alterations of epigenetic regulators, such as DNA methyltransferases, ten-eleven translocation methylcytosine dioxygenases, and histone deacetylase enzymes in different mouse organs and human cell lines [106,107,110,117,118,119]. PFAS-mediated effects on histone modifications and microRNAs have also been described [49,106,107,118,120,121,122].

In summary, epigenetics may play a key role in initiating and maintaining potential pro-cancerous states mediated by non-mutagenic PFAS chemicals. Despite this, very few mechanistic studies have been reported. We speculate that epigenetic reprogramming by PFAS may be driven, in part, by metabolomic alterations in substrates and cofactors of epigenetic enzymes and, reciprocally, that epigenetic-mediated, transcriptional reprogramming plays a key role in establishing and stabilizing the metabolic and hormonal states required for continued tumorigenesis [123,124,125,126,127] (Figure 2). This hypothesis is motivated by the above-mentioned association between PFAS and metabolic, epigenetic, and endocrine disruptions and the recent appreciation of mechanistic relationships between these three pathways. In the following section, we highlight these principles with two cancers possessing epidemiologic links to PFAS: prostate cancer, which is strongly associated with metabolic disruption, and testicular cancer, which is strongly associated with epigenetic reprogramming.

## 3. The Case for Testicular Cancer

There is mounting evidence that testicular germ cell tumors (TGCTs) are especially driven by epigenetics and environmental exposures, including estrogenic exposures. This, coupled with recent epidemiologic evidence linking testicular cancer to PFOA, suggests that TGCTs may be a cancer type especially sensitive to PFAS exposure.

TGCTs are the most common solid cancers of males aged 15–35 [128]. Testicular cancer is a disease of developmental origin, with evidence suggesting that they arise from aberrant primordial germ cells in utero [129]. TGCTs may be especially driven by epigenetics since they have a very low mutational rate compared to other solid tumors, and most patients lack the so-called “driver” mutations found in almost all other solid tumors [130,131]. There is also a link between environmental exposures, for example, estrogenic exposures in utero and early development, and TGCT incidence [132,133,134]. Further, the incidence of TGCTs has greatly increased in industrial nations in the past 50 years, consistent with the premise that exposure to toxic chemicals has impacted TGCT incidence [128]. Epidemiologic studies have indicated that the fetal gonads may be especially sensitive to pro-estrogenic and anti-androgenic insults [132,133,134]. For example, a meta-analysis of 10 studies on EDCs and testicular cancer risk concluded that maternal exposure, but not adult exposure, to EDCs was associated with a >2-fold higher risk of testicular cancer in offspring [132]. This has led to the proposition that testicular cancer is an extreme case of a “testicular dysgenesis syndrome” that includes cryptorchidism, hypospadias, poor semen quality, and male subfertility due to environmental abnormalities, especially those associated with low androgen levels during gonadal development [135,136]. In fact, the above-mentioned conditions, along with congenital disorders of sex development, are known risk factors for TGCTs [134,137,138]. Hence, TGCT etiology matches well with some of the most-studied mechanisms of PFAS action, namely, epigenetics and endocrine disruption. Supporting the idea that TGCTs may be especially sensitive to epigenetic perturbations, we recently found that the polycomb pathway and DNA methylation are interconnected epigenetic drivers of cisplatin sensitivity, resistance, and tumorigenicity in TGCT cells [134,139,140].

Of all cancers, testicular cancer has one of the strongest epidemiological links to PFAS exposure, including in cohort and ecological/case-control studies [13,14,24,25,32]. In the C8 Health Project Dupont plant study of individuals in a community exposed from 1950 to 2004, the incidence of testicular cancer increased with increasing PFOA serum levels, with a 3-fold higher risk in the most-exposed group [24]. TGCTs are one of the eight cancers that PFAS-exposed firefighters contract more often than the general public [141]. In addition, several studies in mice and humans suggest an increase in male reproductive toxicities after prenatal, childhood, adolescent, and adult PFAS exposures [33,34,35]. These include adverse effects on semen quality and quantity, and reproductive hormone levels, which are known to be risk factors for human TGCTs [142,143,144]. While some epidemiological studies specifically concerning PFAS exposure and decreased testosterone levels are conflicting, findings are generally consistent for cohorts exposed in utero, suggesting that the window of exposure is especially critical for PFAS effects on male reproductive health [75,92,93,94]. The strong association between male subfertility and TGCT risk suggests the presence of common etiologic factors. Hence, the testis may be especially vulnerable to EDCs during certain, as yet undefined, windows of susceptibility.

Studies in rats show that PFAS accumulate in the testis, and there is supportive data indicating testicular damage following PFAS exposure [145,146]. PFOS and PFOA exposure in mice and rodents, including in utero exposure, leads to impaired Leydig cell function and in some cases, Leydig cell tumors, both of which are associated with decreased testosterone levels [78,81,84,145,146,147,148,149,150,151]. While some data are also conflicting, as they pertain to PFAS and decreased testosterone in rodents [78,152,153], the data are again more consistent for in utero exposure [81,148,149]. This same trend is also apparent for decreased sperm counts and altered spermatogenesis for PFAS-exposed mice and rats [83,149]. There is also a connection between TGCTs and PPARα, another proposed mechanism of PFAS action. In rodent models, PFAS exposure is known to increase liver expression of CYP19A1, through activation of PPARα resulting in increased estrogen and decreased testosterone levels [43,154]. There is also evidence of a direct effect of PFAS on Leydig cells, leading to deceased production and secretion of testosterone [147].

In summary, epidemiology and experimental evidence suggest that TGCTs may be a key tumor type with which to begin understanding the mechanistic details of epigenetic and endocrine-mediated carcinogenesis as potentially mediated by the PFAS class of environmental toxicants, which may also be relevant to other toxicants.

## 4. The Case for Prostate Cancer

There is evidence associating all three of the major outlined PFAS pathways with the potential promotion of prostate cancer. Prostate cancer and benign prostate cells are dependent on androgens and modulated by other hormones. Hence, it is possible that EDCs could modulate prostate cancer cell homeostasis, leading to prostate cancer progression. Several other EDCs, including cadmium, dioxin, polychlorinated biphenyls, and bisphenol A, have also been associated with prostate cancer progression [155]. PFAS exposure has been shown to potentially increase the risk of prostate cancer in some settings, including for men working in or living near chemical production plants, especially in individuals with a family history of prostate cancer [13,22,23,24,25,26,156].

In addition to environmental and occupational exposures, lifestyle factors, including diet and body weight that alter lipid metabolism, dictate overall increased prostate cancer risk [157,158,159,160]. There is evidence from human prostate cell lines and transgenic mouse models that a high-fat diet contributes to prostate cancer progression by shifting the prostate metabolome to a pro-cancerous state [159,161]. Of note, these actions are mediated, in part, through PPARα, providing the potential for enhanced tumor promotion. We recently showed that PFOS exposure and a high-fat diet synergize to increase prostate cancer xenograft growth in mice [122,162]. PFOS treatment increased glucose metabolism and pyruvate production in prostate cancer cells [122]. In addition, we demonstrated that an enhancement of glycine and serine metabolism and enhanced glucose metabolism, through the Warburg effect in human prostate stem-progenitor cells, took place in response to PFOA and PFOS exposures [162]. Prostate stem-progenitor cells also express PPARα and retinoid X receptor-α which mediate PFAS effects in other tissues [162]. This suggests that PFAS exposure may synergize with a high-fat diet to activate PPARα, resulting in altered cell metabolism to potentially promote tumorigenesis in normal prostate and prostate cancer cells.

The metabolic status of cancer cells determines phenotypic characteristics and drug responses of hormone-dependent cancers [163,164]. Published studies demonstrate that metabolic changes impact epigenetic marks during tumor progression [165,166,167]. Furthermore, PPARα itself is subject to control by epigenetic markers, providing another crosstalk mechanism between metabolism and epigenetics in regulating PFAS actions [21,60]. Metabolic alterations in cancer cells result in epigenetic reprogramming due to changes in the availability of substrates for epigenetic enzymes [123,165,166,167,168,169]. For example, local acetyl-CoA production, via recruitment of metabolic enzymes to chromatin, enables coordination of environmental cues with histone acetylation and gene transcription, which may increase the fitness and survival of cancer cells [168,169]. Reciprocally, epigenetic reprogramming is a common way for cancer cells to adapt to a hostile metabolic environment, mediating inheritable changes in cellular metabolism by altering levels and activity of metabolic regulators [123,124,125,126,127].

## 5. Conclusions

Exposure to PFAS may have adverse, cancer-related health effects, although data from animal models and epidemiology studies are not entirely consistent or conclusive, and many diverse mechanisms of carcinogenicity have been proposed. We contend that three major pathways or properties of PFAS underlie the majority of these mechanisms (Figure 2). Metabolic disruption due to PPAR-dependent and -independent FA mimicry could lead to downstream effects on endocrine homeostasis and epigenetic priming. In turn, epigenetics can provide inheritable and sustainable reprogramming of metabolism and gonadal signaling. Finally, endocrine disruption mediated by PFAS can potentially result in far-reaching, hormone-mediated modulations of both the epigenome and the metabolome. These three interconnected and mutually enforcing pathways may combine to establish a pro-tumorigenic environment for cancer promotion. Notably, each of these pathways is predicted to be highly sensitive to dose, with the potential to be biphasic, and also highly dependent on the window of exposure during the human life cycle, which may explain the sometimes inconsistent epidemiologic and experimental evidence linking PFAS and cancer. These challenges must be met to fully understand the impact of PFAS on cancer development.
